# Vertebral Artery Thrombosis in Chronic Idiopathic Thrombocytopenic Purpura

**DOI:** 10.1155/2017/3184346

**Published:** 2017-06-11

**Authors:** Zakaria Hindi, Nirmal Onteddu, Christopher A. Ching, Abdallah A. Khaled

**Affiliations:** ^1^Department of Internal Medicine, Texas Tech University Health Sciences Center at the Permian Basin, Odessa, TX, USA; ^2^School of Medicine, Texas Tech University Health Sciences Center at the Permian Basin, Odessa, TX, USA; ^3^School of Medicine, University of Jordan, Amman, Jordan

## Abstract

**Introduction:**

Immune thrombocytopenic purpura (ITP) is an autoimmune hematological disorder that causes decreased production and destruction of platelets leading to thrombocytopenia. Although thrombocytopenia usually causes hemorrhagic problems, thrombotic events like strokes, although rare, can still occur. Management of thrombotic events in patients with ITP differs from that of patients with normal platelet count function and count.

**Case Description:**

A 32-year-old female with a history of ITP presented with ischemic stroke. The patient was treated in the hospital with IV immunoglobulin, discharged to a rehabilitation facility, and had complete resolution of symptoms when examined at a follow-up visit 3 months later.

**Conclusion:**

Although stroke in patients with ITP is very rare due to thrombocytopenia, it has been reported in several other published cases and is likely associated with increased platelet microparticle levels, a byproduct of platelet destruction. While usage of antiplatelet therapy in such patients is debated, immunosuppression therapy has been the mainstay treatment in all published cases.

## 1. Introduction

Idiopathic thrombocytopenia purpura is an autoimmune hematological disorder characterized by the destruction of the platelets, which is mediated through antibodies that bind to platelet specific antigens. The antibody-platelet antigen complex is subsequently removed in the spleen by macrophages. If untreated, the continued destruction and removal by spleen can lead to severe thrombocytopenia and severe bleeding. Although bleeding is the usual complication, thrombotic events including cerebrovascular accidents may rarely occur [1, 2]. Thereby, we would like to report a case of female with chronic ITP who developed left vertebral artery thrombosis with left cerebellar infarction.

## 2. Case Description

**Figure 1:**
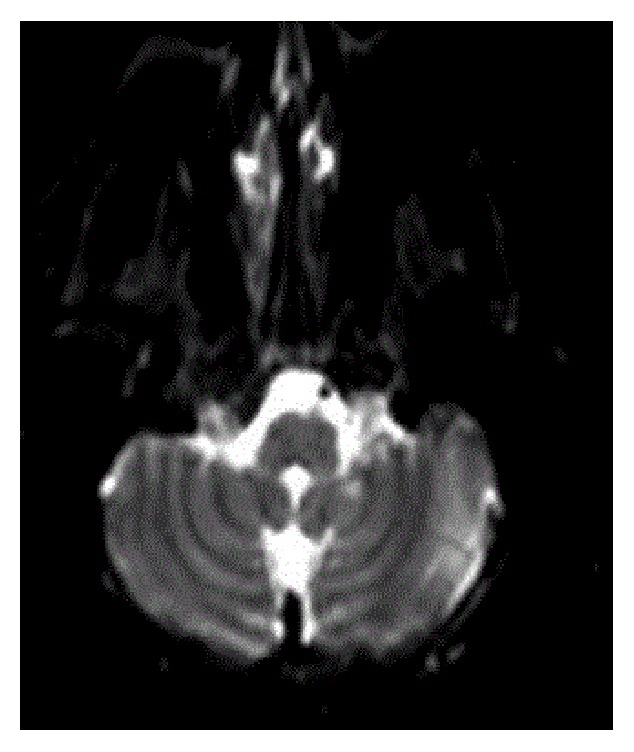
MRI of head on admission, showing multiple infarctions in the left cerebellum.

**Figure 2:**
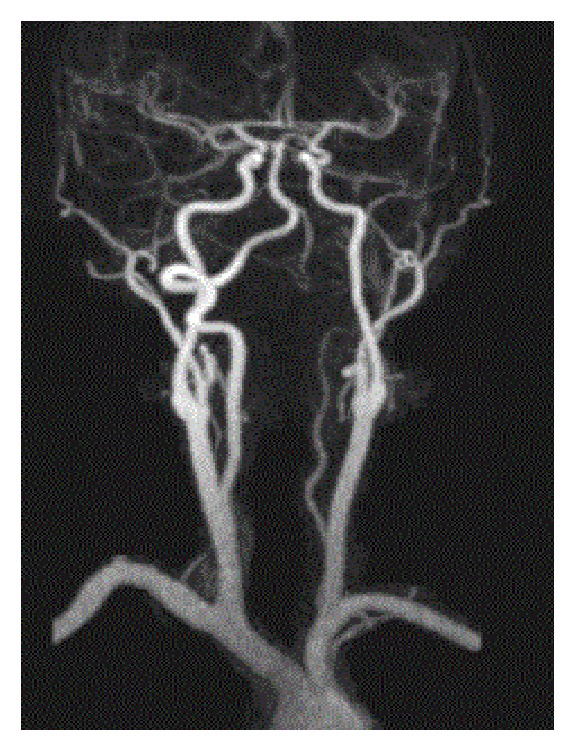
MRA of head and neck on admission, showing incomplete occlusion of left vertebral artery.

**Figure 3:**
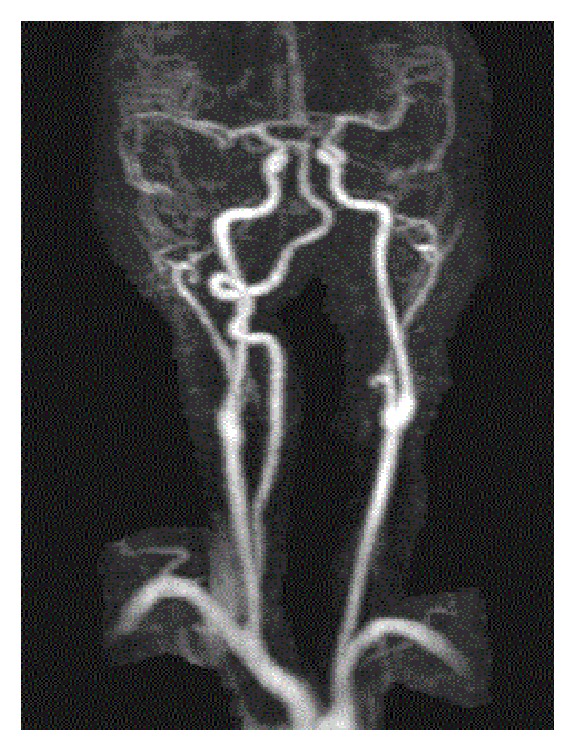
MRA of head and neck at 3-month follow-up visit.

**Figure 4:**
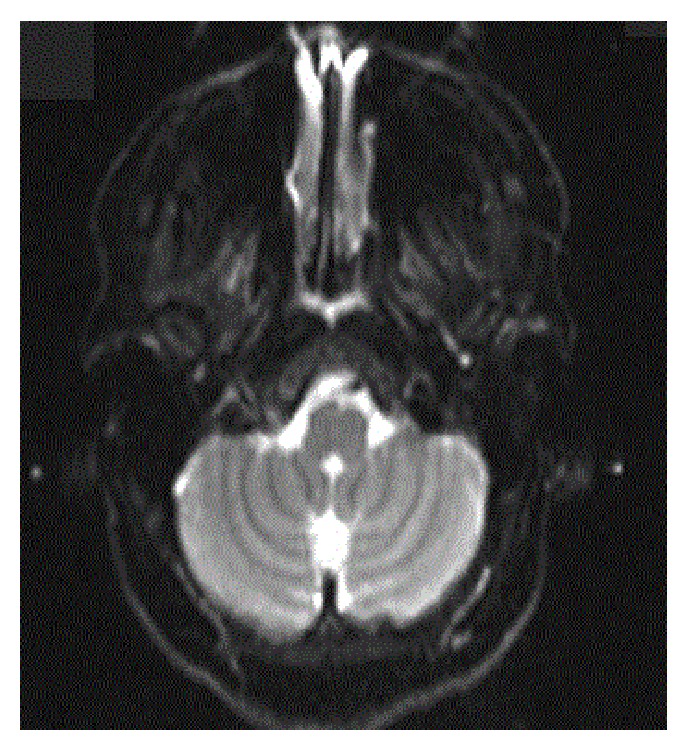
MRI of head and neck at 3-month follow-up visit.

A 32-year-old female with a history of chronic idiopathic thrombocytopenia purpura (ITP) presented to emergency room with vertigo and imbalance for two hours prior to admission. Physical examination was remarkable for cerebellar ataxia, positive Romberg's sign, dysmetria, and dyskinesia on the left upper limb. Urgent CT scan of head was done and showed no signs of intracranial hemorrhage. Complete blood count (CBC) on admission was only remarkable for platelet count of 49 K/uL. The rest of blood work-up including coagulation profile and comprehensive metabolic panel was unremarkable. The decision was made to start the patient on intravenous immunoglobulins (IVIG) while investigating for cerebrovascular accident. MRI for head and neck was done and showed infarction areas in left cerebellar hemisphere (Figure 1 (Radiology Department at Hamad General Hospital, Doha, Qatar)). MRA (Figure 2 (Radiology Department at Hamad General Hospital, Doha, Qatar)) for head and neck was done as well, which showed an occlusion in the distal segment of left vertebral artery. Transthoracic echocardiogram showed no thrombus formation in left atrium. Further investigations including factor V Leiden mutation, proteins C and S activity, complement 3 and 4 levels, anti-phospholipid antibodies, anti-nuclear antibody, and antithrombin 3 level were all unremarkable. After consulting hematology and neurology team, the decision was to avoid both anticoagulant therapy for venous thromboembolism and antiplatelet therapy and to continue IVIG for five days total. One week later, the patient's symptoms improved with some residual difficulty walking. Average platelets count in the one-week period was 45 K/uL. The patient was transferred to a rehabilitation facility, where she continued to improve and was discharged afterwards. Three months later, in a follow-up visit, MRA for neck showed complete occlusion of left vertebral artery (Figure 3 (Radiology Department at Hamad General Hospital, Doha, Qatar)). MRI for head showed complete resolution of the cerebellar infarction (Figure 4 (Radiology Department at Hamad General Hospital, Doha, Qatar)). CBC was obtained during the visit and her platelet count was 46 K/uL. The patient was completely asymptomatic and was advised to avoid antiplatelet therapy.

## 3. Discussion

Eight published cases of ITP with ischemic stroke were found after reviewing the literature, none of which described vertebral artery thrombosis. The sites of the infarction in seven of the cases were in cortical regions [3–9] and only one with thalamic infarction [10]. Out of the reports with cortical infarctions, only two were involving the posterior circulation (occipital lobes) [6, 9]. The etiology of ischemia in these cases seemed to be thrombotic rather than embolic, as there were no risk factors that favored embolic events (Table 1 summarizes the main characteristics of ITP patients [3–10]).

The association between thrombosis and ITP is still not well established. However, evidence has shown that platelet microparticles (PMP) have a major role in thrombogenesis in ITP. PMP is a substance that is produced from platelets upon destruction. Studies have shown that the levels of PMP are higher in both ITP and non-ITP patients with cerebral ischemic changes compared to healthy individuals. Furthermore, the high level PMP was found to be protective against hemorrhage. However, in excess levels, PMP can promote thrombin formation and thus thrombosis [11–14]. Other mechanisms of thrombogenesis in ITP are the treatment with IVIG. IVIG can promote thrombosis by increased blood viscosity and increased thrombin production and by directly affecting vascular endothelium with associated cerebral arterial vasospasm [15–18].

The treatment of ITP ischemic stroke is controversial as both hematologist and neurologist have different perspectives. All mentioned cases have used immunosuppression therapy (IVIG, steroids, or cyclosporine). The importance of immunosuppression in the setting of ITP and ischemic stroke likely is due to the resulting decrease of PMP levels, as mentioned by Ichijo et al. [7]. As for antiplatelet therapy, the decision varied among the cases. In two of them, antiplatelet therapy was given and there were no hemorrhagic events [4, 7]. On the other hand, antiplatelet therapy was withheld in three cases without any further adverse thrombotic events [5, 8, 10]. Other treatments such as calcium channel blockers were given in two cases. Since platelet fragmentation is calcium dependent, these agents can prevent the release of PMP by inhibiting extracellular calcium influx [13, 14].

## 4. Conclusion

Treatment of ischemic stroke in ITP is still controversial; nevertheless, the use of immunosuppression modality and consulting both hematology and neurology teams are essential for initial plan of care.

## Figures and Tables

**Table 1:** Summary of published cases (ITP: idiopathic thrombocytopenia purpura, HTN: hypertension, and APS: antiphospholipid syndrome).

Author of reported cases	Age of the patient	Gender	Site of infarction	Risk factors
Otsuki et al.	38	Female	Left temporal and parietal regions	APS and ITP
Gümüş and Yılmaz	55	Female	Left middle cerebral artery territory	ITP
Hashmi	57	Male	Right middle cerebral artery territory	ITP
Theeler and Ney	63	Male	Right occipital lobe infarction	ITP
Peña et al.	84	Male	Left thalamic infarction	ITP
Ichijo et al.	60	Female	Middle cerebral artery territory	ITP
Mahawish et al.	79	Male	Multiple focal ischemic lesions in both cerebral hemispheres	HTN and ITP
Nanri et al.	31	Male	Right occipital lobe and hippocampal gyrus	Migraine headaches and ITP
